# Supplementary material for: Content-rich biological network constructed by mining PubMed abstracts
Source: BMC Bioinformatics. 2004 Oct 8;5:147. doi: 10.1186/1471-2105-5-147 (PMC528731; doi:10.1186/1471-2105-5-147)
Supplement: Additional File 5 — The original Chilibot query results of the term "long-term potentiation (LTP)" and 22 other terms, limiting the latest references analyzed to the years 1990, 1995, 2000, and 2004. [file 1471-2105-5-147-S5.bz2 › chilibotAdditionalFile5/ltp2004/html/LTP_ZIF268.html]

 


 **LTP** and **ZIF268** 
  
Found 20 abstracts in PubMed,  **20 abstracts were retrieved and analyzed**.  


---

 Search Google  |
 PDF files only 
|  EDU domain only 

---

**Interactive relationship** (e.g. stimulation, inhibition, etc)

- These results establish the existence of subfield specific transcriptional responses to  **LTP**  inducing stimulation in the hippocampus of the intact animal, and demonstrate that in area CA1 enhanced transcription of  **Zif268** , Arc and Homer is not required for the induction of late  **LTP** .  Ref: 11264669 Eur J Neurosci, 2001
- The induction of long term potentiation  [ **LTP** ]   **LTP**  in the dentate gyrus of the hippocampus is associated with a rapid and robust transcription of the immediate early gene  **Zif268** .  Ref: 11224546 Nat Neurosci, 2001
- The extent of  **LTP**  saturation in fascia dentata produced by the standard chronic  **LTP**  stimulation protocol was assessed both electrophysiologically and through the use of an anatomical marker activation of the immediate early gene  **zif268** .  Ref: 7931545 J Neurosci, 1994
- Several studies have shown that  **zif268**  mRNA is upregulated during different forms of associative learning, and following tetanic stimulation that induces long lasting  **LTP** .  Ref: 12798262 Behav Brain Res, 2003
- We have compared changes in mRNA of three genes,  **zif268** , raf B, and syntaxin 1 B, following the unilateral induction of long term potentiation  [ **LTP** ]   **LTP**  in rats previously trained in a water maze, and in behaviourally naive animals.  Ref: 9714460 Neurosci Lett, 1998
- In granule cells of the dentate gyrus,  **LTP**  induced in vivo by tetanic stimulation of the perforant path was followed by strong induction of the immediate early genes IEGs  **Zif268** , Arc and Homer.  Ref: 11264669 Eur J Neurosci, 2001
- These results suggest that  **LTP**  and spatial training engage a common pathway utilizing an increase in mRNA for raf B, and demonstrate a dissociation between  **LTP**  and spatial learning with respect to expression of  **zif268**  and syntaxin 1B.  Ref: 9714460 Neurosci Lett, 1998
- Three of these genes were known only from expressed sequence tags RM1 3, two were known genes whose up regulation by  **LTP**  has not been described GADD153 CHOP and ler5, and five were known genes whose up regulation by  **LTP**  has already been reported MAPK phosphatase, NGFI A  **zif268** , vesl 1S homer 1a, Ag2, and krox 20.  Ref: 10820183 J Neurochem, 2000
- Here, we briefly review experiments that have shown MAPK ERK, cAMP response element binding protein CREB and the immediate early gene IEG  **zif268**  are essential components of a signalling cascade required for the expression of late phase  **LTP**  and of certain forms of long term memory.  Ref: 12740127 Philos Trans R Soc Lond B Biol Sci, 2003

**Parallel relationship** (e.g. studied together, co-existance, homology, etc.)

- We used a mutant mouse with a targeted disruption of  **Zif268**  to ask whether this gene, which encodes a zinc finger transcription factor, is required for the maintenance of late  **LTP**  and for the expression of long term memory.  Ref: 11224546 Nat Neurosci, 2001
- At the age when these animals developed cognitive dysfunction, they had reduced mRNA expression of several genes essential for long term potentiation  [ **LTP** ]  and memory formation Arc,  **Zif268** , NR2B, GluR1, Homer 1a, Nur77 TR3.  Ref: 12832546 J Neurosci, 2003
- In contrast, we were unable to detect modulation of the IEGs  **Zif268** , Arc, Homer and HB GAM following induction of  **LTP**  by high frequency stimulation of the commissural projection to CA1 pyramidal cells in vivo.  Ref: 11264669 Eur J Neurosci, 2001
- **Zif268** , Homer and syntaxin 1B genes were studied, and their regulated expression was examined three times after the induction of long term potentiation  [ **LTP** ] .  Ref: 11958865 Neuroscience, 2002
- In contrast, administration of maximal electroconvulsive shock led to robust  **zif268**  activation throughout the hippocampus, enhancement of synaptic responses, occlusion of  **LTP**  produced by discrete high frequency stimulation, and spatial learning deficits in the water task.  Ref: 7931545 J Neurosci, 1994
- A requirement for the immediate early gene  **Zif268**  in the expression of late  **LTP**  and long term memories.  Ref: 11224546 Nat Neurosci, 2001
- Finally, we investigated the expression of two immediate early genes, activity regulated cytoskeleton associated protein and  **Zif268** , both of which are required for generation of late, mRNA synthesis dependent  **LTP** .  Ref: 11880483 J Neurosci, 2002
- When  **LTP**  was induced in vitro neither CA1 pyramidal cells nor granule cells showed an increase in  **Zif268** , Arc or Homer mRNA.  Ref: 11264669 Eur J Neurosci, 2001
- We also show that ERKI II is necessary for the upregulation of the proteins encoded by the immediate early genes  **Zif268**  and Homer after the induction of  **LTP**  in the dentate gyrus by tetanic stimulation of the perforant path in vivo or by BDNF stimulation of primary cortical cultures.  Ref: 12097495 J Neurosci, 2002
